# Profound Hypokalaemia Resulting in Maternal Cardiac Arrest: A Catastrophic Complication of Hyperemesis Gravidarum?

**DOI:** 10.1155/2018/4687587

**Published:** 2018-07-29

**Authors:** Anna Walch, Madeline Duke, Travis Auty, Audris Wong

**Affiliations:** ^1^Doctor of Medicine (MD), Griffith University, Australia; ^2^Lecturer, School of Medicine, Griffith University, Australia; ^3^Obstetrics and Gynaecology Principal House Officer, Gold Coast University Hospital, Australia; ^4^Bachelor of Medicine and Bachelor of Surgery (MBBS), Bond University, Australia; ^5^Obstetric Medicine Fellow, Royal Brisbane and Women's Hospital, Australia; ^6^Endocrine Advanced Trainee, Royal Brisbane and Women's Hospital, Australia; ^7^Bachelor of Medicine and Bachelor of Surgery (MBBS), Griffith University, Australia; ^8^Intensive Care Unit Registrar, Gold Coast University Hospital, Australia; ^9^Obstetrics and Gynaecology Staff Specialist, Gold Coast University Hospital, Australia; ^10^FRANZCOG (Fellowship of the Royal Australian and New Zealand College of Obstetricians and Gynaecologists), Australia; ^11^MRCOG (Membership of the Royal college of Obstetricians and Gynaecologist), UK; ^12^MRCPI (Membership of the Royal College of Physicians in Ireland (Obstetrics and Gynaecology and General Medicine)), Ireland

## Abstract

We present a case of a 39-year-old G8P6 Pacific Islander woman who at 15+5 weeks' gestation had an out-of-hospital cardiac arrest secondary to profound hypokalaemia which was associated with severe hyperemesis gravidarum (HG). Her clinical course after arrest was complicated by a second 5-minute cardiac arrest in the Intensive Care Unit (ICU) (pre-arrest potassium 1.8), anuric renal failure requiring dialysis, ischaemic hepatitis, and encephalopathy and unfortunately fetal demise and a spontaneous miscarriage on day 2 of admission. Despite these complications, she was discharged home 4 weeks later with a full recovery. Following a plethora of inpatient and outpatient investigations, the cause of her cardiac arrest was determined to be profound hypokalaemia. The hypokalaemia was presumed second to a perfect storm of HG with subsequent nutritional deficiencies causing electrolyte wasting, extracellular fluid (ECF) volume reduction, and activation of the renin-angiotensin-aldosterone axis (RAAS). This combined with the physiological changes that promote potassium wasting in pregnancy including volume expansion, increased renal blood flow, increased glomerular filtration rate, and increase in cortisol contributed to the patient having a profoundly low total body potassium level. This diagnosis is further strengthened by the fact that her pre- and post-pregnancy potassium levels were within normal limits in the absence of supplementary potassium. This case highlights the potentially life-threatening electrolyte imbalances that can occur with HG and the importance of recognising the disease, comprehensive electrolyte monitoring, and aggressive management in pregnancy.

## 1. Background

Hyperemesis gravidarum (HG) is a severe form of nausea and vomiting that affects 0.3–3% of pregnancies [1]. It is characterised by severe, protracted nausea and vomiting associated with weight loss of more than 5% of pre-pregnancy weight, dehydration, and electrolyte imbalances [2, 3]. HG can be associated with significant morbidity including pneumomediastinum, renal failure, liver dysfunction, Boerhaave's syndrome, and Wernicke's encephalopathy [1]. In the fetus, it is also associated with an increased risk of adverse pregnancy outcomes including low birth weight, neurodevelopmental disorders, intrauterine growth restriction, preterm delivery, and fetal and neonatal death [4]. Although maternal death is a rare complication of HG, there are still reported cases of it annually in first-world countries [4]. This is the first case report detailing a case of cardiac arrest from hypokalaemia second to HG and presents the critical importance of proactively managing nutritional and metabolic imbalances associated with HG.

## 2. Case

We present a case of a 39-year-old G8P6M1 Pacific Islander woman who at 15+5 weeks gestation was brought in by ambulance following an out-of-hospital cardiac arrest. The arrest was witnessed by her family at home who contacted the ambulance service and commenced cardiopulmonary resuscitation (CPR). She was resuscitated at the scene involving CPR with approximately 40 minutes downtime, cold intubation, and multiple direct current cardioversions for stabilisation. On arrival to the emergency department she had fixed dilated pupils and was found to be significantly acidotic (pH 6.7, lactate 26mmol/L) with associated hypokalaemia of 2.1mmol/L (range 3.5-5.2mmol/L) (see Tables 1 and 2). Her initial resuscitation and stabilisation involved a potassium infusion up to 40 mmol/hr and an adrenaline and noradrenaline infusion, 4 units of packed red blood cells, and 4 units of albumin. Following stabilisation and electrolyte repletion, she had a second 5-minute ventricular fibrillation (VF) arrest 4 hours later in the ICU, where her potassium on her preceding venous blood gas was 1.8mmol/L. She was commenced on 300mg of IV Thiamine daily from day one of her ICU admission. On day one of admission, the pregnancy was still viable with a FHR of 150beats per minute detected. Unfortunately, on day two of her admission, there was fetal demise and she spontaneously miscarried in the ICU and required a dilatation and curettage for retained products of conception. Her inpatient stay was complicated by multiorgan dysfunction including ischaemic hepatitis, mild encephalopathy requiring rehabilitation, and anuric renal failure requiring short-term dialysis.

The patient's pregnancy history was unremarkable preceding the out-of-hospital cardiac arrest except for an early positive oral glucose tolerance test (OGTT) (in the absence of evidence of type 2 diabetes mellitus with a normal HbA1C) performed at 13 weeks' gestation (see Table 3). On the day of her arrest her husband did not notice any additional symptoms and her nausea and vomiting did not particularly worsen, but she did manage to tolerate a small lunch meal prior to arresting. She had a background history of 5 previous pregnancies to the same partner, complicated by some nausea and vomiting in those pregnancies, with no definitive evidence of HG. In this pregnancy, from an early gestation, the patient confirmed the presence of significant nausea and vomiting, with emesis occurring after every meal on most days. She had limited oral intake as a result and ensuing weight loss occured with approximately 10kg's lost in total (9% of total body weight).

The patient's background medical history was otherwise unremarkable with no symptoms or biochemical evidence of a disorder of potassium homeostasis preceding the pregnancy. Specifically, she denied symptoms to suggest hypokalaemic periodic paralysis and serial serum electrolyte testing revealed normal potassium levels before and after pregnancy (see Table 1). Urine electrolyte testing was also unremarkable postpartum, with no evidence of renal potassium wasting (see Table 4). Furthermore, she had no history to suggest an arrhythmogenic disorder with no palpitations, presyncope, or syncope reported. She had no significant family history of cardiomyopathy or sudden cardiac death and no personal history of valvular heart disease or rheumatic fever. She denied symptoms to suggest thyroid disease and pre-pregnancy had normal Thyroid Stimulating Hormone (TSH) levels on serial testing, including a normal TSH level at 7-weeks gestation. She was not hypertensive, and there was no evidence of primary hyperaldosteronism on testing. There was no clinical evidence of cortisol excess, and screening 24-hour urinary free cortisol was normal. She was not on any regular medications and had no known drug allergies.

The patient was thoroughly investigated for causes and contributors to her cardiac arrest. Results of cardiac investigations included a normal coronary angiogram, normal left ventricle ventriculogram, and a transoesophageal echocardiogram which demonstrated preserved biventricular systolic function and structurally normal valves with moderate mitral regurgitation. She had a normal computed tomography pulmonary angiogram (CTPA) with no evidence of pulmonary embolism. She had a largely normal CT head with a 10mm filling defect in her left transverse sinus but nil other acute pathology. She had a normal baseline electrocardiogram (ECG) post-arrest with no evidence of long QT syndrome.

Following stabilisation and correction of her potassium (see Table 1) and acute renal failure, she was discharged home after a total 33-day admission. She transitioned well to home where she is continuing to care for her children, the youngest of which is 3 years old. She is independent with her activities of daily living and mobility and only suffered from mild short-term memory impairment and mild impairment in her concrete problem solving. Importantly, her serum potassium level remains normal.

## 3. Discussion

A case of maternal cardiac arrest second to profound hypokalaemia in a patient with HG presents a unique opportunity to discuss the potential implications of this common disorder. A patient with HG frequently vomits gastric juice and, thus, the loss of hydrogen ions, sodium, chloride, and water in gastric contents leads to chloride-sensitive metabolic alkalosis, dehydration, and extracellular fluid (ECF) volume reduction [5]. Our patient had lost approximately 9% in body weight and likely was volume deplete, causing elevated activity of the renin-angiotensin-aldosterone system (RAAS) [5]. This activated RAAS, in turn, increases the urinary excretion of potassium, compounding the hypokalaemia [6]. This severe hypokalaemia eventually led to her sudden cardiac arrest. On presentation, her potassium was low at 2.1mmol/L. This was an unexpected finding in a patient post-cardiac arrest. It is more common after arrest to see hyperkalaemia due to the exchange of potassium ions with hydrogen ions and the movement of intracellular potassium to the extracellular space in an attempt to correct the acidosis [6]. It was presumed that her hypokalaemia was much more severe prior to her arresting.

Many of the reported serious morbidity and even mortality second to HG in the literature have presented with Wernicke's encephalopathy (WE) or associated thyrotoxicosis. There are increasing reports of maternal mortality secondary to WE from HG which is caused by thiamine (vitamin B1) deficiency [7, 8]. WE typically manifests with nonspecific symptoms of headache, confusion, and irritability but then progresses to spastic paresis, ataxia, oculomotor dysfunction, myoclonus, and nuchal rigidity [7, 8]. Rarely, thiamine deficiency can cause cardiovascular compromise which is termed “wet beriberi” which could eventually lead to cardiac arrest [9]. Wet beriberi presents with clinical signs of congestive cardiac failure including tachycardia, dyspnoea, peripheral oedema, and cardiomegaly with normal sinus rhythm [9]. Our patient and her family denied any presence of these signs and symptoms as well as no preceding symptoms of WE including weakness, confusion, or ataxia. After arrest, our patient had no significant abnormalities on echocardiogram suggesting no involvement of thiamine deficiency as a cause for her cardiovascular compromise. Macgibbon reports a mortality of a pregnant patient who had respiratory failure second to HG, hypokalaemia, and Wernicke's encephalopathy [8]. That patient, however, presented with symptoms of slurred speech, confusion, and weakness and subsequently later died from rapid correction of hyponatremia causing osmotic demyelination syndrome and respiratory arrest [8]. However, given her history of HG, our patient was treated with 300mg of IV thiamine daily from admission which is the gold standard treatment for thiamine deficiency and WE.

There is a case report by Iwashita [10] that attributes hypokalaemia second to HG as a cause of arrest and maternal death. The authors report on an obese pregnant woman who suffered a respiratory arrest at 12+4 weeks' gestation that was attributed to severe HG causing profound hypokalaemia. They attributed the respiratory arrest to severe potassium deficiency causing respiratory muscle paralysis and this was compounded by obesity [10].

There have been several case reports of refeeding syndrome causing severe hypokalaemia in pregnancy. Refeeding syndrome is usually seen in the context of increased caloric intake after prolonged periods of malnutrition. For example, Majumdar reports a case of refeeding syndrome after treatment of severe HG with NJ feeds [11]. Refeeding syndrome manifests as severe hypophosphataemia, hypoklaemia, hypomagnesaemia, fluid retention, and altered glucose homeostasis [11]. Against this diagnosis, our patient presented with marked hyperphosphataemia (4.95mmol/L) and hypermagnesaemia (1.25mmol/L) which is inconsistent with the electrolyte derangement that are the hallmarks of refeeding syndrome [12, 13].

Fezjo et al. report that five out of six patients in their case series who died from complications secondary to HG, presented with hypokalaemia [4]. Therefore, patients who present with HG accompanied by hypokalaemia may represent a high-risk subgroup that should be closely monitored and treated until complete and prolonged stabilisation of potassium and other electrolyte levels is achieved. The importance of potassium homeostasis is highlighted by the finding that patients with potassium abnormalities have an increased rate of death from any cause [4].

First it must be highlighted that although the most likely cause of hypokalaemia in this case was hyperemesis; some rare differentials cannot be completely excluded. One is hypokalaemic periodic paralysis, which could be exacerbated by undiagnosed hyperthyroidism in pregnancy [14]. This cannot be completely disproved as there were no thyroid function tests (TFTs) performed at the time of the cardiac arrest. However, the fact that TFTs earlier in the pregnancy and subsequent TFTs shortly after the miscarriage were normal (*T4 9.8pmol/L ref 7.0-17, T3 5.4pmol/L ref 3.5-6.0 and TSH 1.1mU/L ref 0.3-4.5*) makes this diagnosis highly unlikely. Additionally, the patient reported no previous history to suggest periodic paralysis, even though this condition is more common in people of Pacific Islanders descent [15].

Other rare differential diagnoses include a tubulointerstitial disorder resulting in excessive renal potassium wasting. One example of such a disorder is Gitelman's syndrome [5]. This is extremely unlikely given that she had normal urinary electrolytes with no evidence of sodium or potassium wasting outside of pregnancy and no personal or family history to suggest this [16]. But it has been considered that the patient could excessively waste urinary potassium in pregnancy exclusively.

## 4. Conclusion

This is a unique case of profound hypokalaemia in pregnancy resulting in maternal cardiac arrest. The most likely aetiology was gestational emesis and gestational diabetes mellitus which aggravated the normal potassium wasting of pregnancy; however, it is impossible to exclude all potential contributors to the development of hypokalaemia in a patient with no preceding or post-event electrolyte derangements. This case highlights the fact that HG can cause profound metabolic and electrolyte disturbances in patients with the potential for resultant catastrophic consequences. Clinicians should consider performing a serum screening of electrolytes in all patients who report persistent severe nausea and vomiting in pregnancy with associated weight loss and appropriately correct the imbalances.

## Figures and Tables

**Table 1:** Serum electrolytes.

	*Pre-pregnancy*	*On Admission*	*On discharge from hospital*
*Potassium K* ^*+*^ * (mmol/L) range 3.5-5.2*	*3.8*	*2.1*	*3.9*

*Magnesium Mg* ^*2+*^ * (mmol/L) range 0.75-1.1*	*0.89*	*1.25*	*0.82*

*Phosphate *P0^4^_3-_* (mmol/L) range 0.75-1.50*	*1.32*	*4.95*	*1.27*

**Table 2:** Venous blood gas (VBG) on arrival to the emergency department.

pH *range 7.35-7.45*	6.71	Haemoglobin (g/L) *range 110-140*	108

pC02 (mmHg) *range 35-45*	102	Sodium Na^+^* (mmol/L) range 135-145*	135

Bicarbonate HCO_3_^−^* (mmol/L) range 22-28*	12	Potassium K^+^* (mmol/L) range 3.5-5.2*	2.1

Anion gap *(mmol/L) range 4-12*	34	Chloride Cl^−^* (mmol/L) range 98-106*	91

Lactate *(mmol/L) range 0.5-2*	26.0	Glucose *(mmol/L) range 3.9-7.5*	22.0

**Table 3:** Oral glucose tolerance test.

*Fasting plasma glucose*	*5.4 mmol/L (range <5.1)*

*1-hour glucose*	*7.5 mmol/L (range <10.0)*

*2-hour glucose*	*7.6 mmol/L (range <8.5)*

**Table 4:** Urinary electrolytes/cortisol (24hr collection).

*Potassium K+*	*49 mmol/24hr (range 25-125)*

*Creatinine*	*12.5 mmol/24hr (range 6.0 – 14.0)*

*Cortisol (free)*	*26 nmol/24hr (range 10 - 120)*

## References

[1] Tan P. C., Jacob R., Quek K. F., Omar S. Z. (2007). Pregnancy outcome in hyperemesis gravidarum and the effect of laboratory clinical indicators of hyperemesis severity. *Journal of Obstetrics and Gynaecology Research*.

[2] Niemeijer M. N., Grooten I. J., Vos N. (2014). Diagnostic markers for hyperemesis gravidarum: a systematic review and metaanalysis. *American Journal of Obstetrics & Gynecology*.

[3] Bustos M., Venkataramanan R., Caritis S. (2017). Nausea and vomiting of pregnancy - What's new?. *Autonomic Neuroscience: Basic and Clinical*.

[4] Fejzo M. S., MacGibbon K. (2016). Why are women still dying from nausea and vomiting of pregnancy?. *Gynecology & Obstetrics Case Report*.

[5] Acelajado M. C., Culpepper R. M., Bolton III W. D. (2016). Hyperemesis gravidarum in undiagnosed Gitelman's syndrome. *Case Reports in Medicine*.

[6] Aronson P. S., Giebisch G. (2011). Effects of pH on potassium: New explanations for old observations. *Journal of the American Society of Nephrology*.

[7] Scalzo S. J., Bowden S. C., Ambrose M. L., Whelan G., Cook M. J. (2015). Wernicke-Korsakoff syndrome not related to alcohol use: a systematic review. *Journal of Neurology, Neurosurgery & Psychiatry*.

[8] MacGibbon K. W., Fejzo M. S., Mullin P. M. (2015). Mortality secondary to hyperemesis gravidarum: a case report. *Women's Health & Gynecology*.

[9] Huertas-González N., Hernando-Requejo V., Luciano-García Z., Cervera-Rodilla J. L. (2015). Wernicke’s encephalopathy, wet beriberi, and polyneuropathy in a patient with folate and thiamine deficiency related to gastric phytobezoar. *Case Reports in Neurological Medicine*.

[10] Iwashita A., Baba Y., Usui R., Ohkuchi A., Muto S., Matsubara S. (2015). Respiratory arrest in an obese pregnant woman with hyperemesis gravidarum. *Case Reports in Obstetrics and Gynecology*.

[11] Majumdar S., Dada B. (2010). Refeeding syndrome: a serious and potentially life-threatening complication of severe hyperemesis gravidarum. *Journal of Obstetrics & Gynaecology*.

[12] Chiarenza L., Pignataro A., Lanza V. (2005). Refeeding syndrome in early pregnancy. Case report. *Minerva Anestesiologica*.

[13] Marinella M. A. (2005). Refeeding syndrome and hypophosphatemia. *Journal of Intensive Care Medicine*.

[14] Kung A. W. C. (2006). Clinical Review: thyrotoxic periodic paralysis: a diagnostic challenge. *The Journal of Clinical Endocrinology & Metabolism*.

[15] Elston M. S., Orr-Walker B. J., Dissanayake A. M., Conaglen J. V. (2007). Thyrotoxic, hypokalaemic periodic paralysis: polynesians, an ethnic group at risk. *Internal Medicine Journal*.

[16] Kurtz I. (1998). Molecular pathogenesis of Bartter's and Gitelman's syndromes. *Kidney International*.

